# *Annurca* Apple Polyphenol Extract Affects Acetyl- Cholinesterase and Mono-Amine Oxidase In Vitro Enzyme Activity

**DOI:** 10.3390/ph14010062

**Published:** 2021-01-14

**Authors:** Rosarita Nasso, Valentina Pagliara, Stefania D’Angelo, Rosario Rullo, Mariorosario Masullo, Rosaria Arcone

**Affiliations:** 1 Dipartimento di Scienze Motorie e del Benessere, Università degli Studi di Napoli “Parthenope”, Via Medina 40, 80133 Napoli, Italy; rosaritanasso@gmail.com (R.N.); valentina.pagliara83@gmail.com (V.P.); stefania.dangelo@uniparthenope.it (S.D.); 2 ISPAAM, Consiglio Nazionale delle Ricerche, Piazzale Enrico Fermi 1, 80055 Portici, Italy; rosario.rullo@cnr.it; 3 CEINGE, Biotecnologie Avanzate, S.C. a R.L., Via G. Salvatore 486, 80145 Napoli, Italy

**Keywords:** *Annurca* apple polyphenols, cholinesterase activity, monoamine oxidase activity, neuroblastoma cells, cell viability

## Abstract

In this study, we explored the ability of *Annurca* apple flesh polyphenol extract (AFPE) to affect the activity of key enzymes involved in neurodegenerative disorders—in particular, Acetyl- and Butirryl-cholinesterases, and type A and B monoamine oxidase. The effect of AFPE on enzyme activity was analyzed by in vitro enzyme assays, and the results showed concentration-dependent enzyme inhibition, with IC_50_ values corresponding to 859 ± 18 µM and 966 ± 72 µM for AChE and BuChE respectively, and IC_50_ corresponding to 145 ± 3 µM and 199 ± 7 µM for MAO-A and MAO-B, respectively, with a preference for MAO-A. Moreover, in this concentration range, AFPE did not affect the viability of human neuroblastoma SH-SY5Y and fibroblast BJ-5ta cell lines, as determined by an MTT assay. In conclusion, our results demonstrate that AFPE shows the new biological properties of inhibiting the activity of enzymes that are involved in brain functions, neurodegenerative disorders, and aging.

## 1. Introduction

Natural polyphenols from fruits and vegetables exert beneficial effects on human health due to their antioxidant and anti-inflammatory properties [1,2,3,4]; they also behave as anticancer agents on various tumor cells [5]. Polyphenols are a group of naturally occurring phytochemicals characterized by the presence of multiple hydroxyl groups on aromatic rings, and are divided into two main categories—flavonoids and nonflavonoids—based on the number of phenol rings and the way in which these rings are linked. They are micronutrients present in abundance in our diet, of which the most important sources are fruit and vegetables, red wine, green and black tea, coffee, chocolate, olives, and extra virgin olive oil, but also herbs and spices, nuts, and algae [6]. Among fruits, the apple is the most extensively produced and consumed worldwide, and is one of the richest in polyphenolic compounds [7].

The *Annurca* (Malus pumila Mill, *cv Annurca*) is well known as the “the apple queen” due to its organoleptic qualities, taste, scent, and flavor. This apple is renowned for its white, crisp, and compact flesh, having a pleasantly acidulous and fragrant flavor, making it different from other apple varieties [8]. *Annurca* is an apple with a “Protected Geographical Indication” of the Campania region which accounts for 5% of Italian apple production, is particularly rich in catechin and epicatechin, and is characterized by a stronger antioxidant action than other varieties [9]. Preceding studies from our laboratory have revealed both antioxidant [10,11,12] and pro-oxidant/proapoptotic [12,13,14,15,16,17] effects of *Annurca* flesh polyphenol extract (AFPE) on human cells.

Moreover, natural polyphenols also play a role in brain function, demonstrating neuroprotective activity in both in vitro or in vivo models of neuronal cell death [18,19,20]. Compounds such as resveratrol, curcumin, nuts, blueberry polyphenols, sulforaphanes, salvionic acids, and ω-3 fatty acids (as docosahexaenoic acid) have been shown to induce neurogenesis in the adult brain [21,22,23,24,25].

These compounds affect neurogenesis by different mechanisms that reduce oxidative stress and neuro-inflammation as well as promote pathways involved in autophagy, cell repair, and survival [21,22,23].

Among neurodegenerative disorders, Alzheimer’s disease (AD), characterized by memory loss and learning deficits, is caused by extensive neurodegeneration, neuronal death, and shrinkage of larger areas of the brain. Currently, acetylcholinesterase (AChE) inhibitors are the dominant class of approved drugs to treat AD, and therefore, much effort has been devoted to the identification of novel natural compounds that are able to inhibit AChE as potential drugs for the treatment of this disease [26,27,28]. Among natural compounds, polyphenols from lemon peel extract also show in vitro acetylcholinesterase inhibitory activity [29].

However, AD is a multifactorial disorder that appears to be also correlated to the overexpression of mono-amine oxidase (MAO) enzymes that are involved in brain functions and age-related neuro-degenerative disorders, such as Parkinson’s disease [30]. MAO enzymes catalyze oxidative catabolism of monoamines and regulate the homeostasis of monoamine neurotransmitters in the brain. MAO are classified as type A and B (MAO-A, MAO-B), based on substrate specificity and inhibitor sensitivity [31].

MAO-B is considered to be associated with neuronal death caused by oxidative stress and neurotoxin synthesis as seen in Parkinson’s disease; MAO-B inhibitors, rasagiline and selegiline [(-)deprenyl], protect neurons in animal and cellular models of neurodegeneration [32]. The selective MAO-A inhibitor, clorgylin [N-methyl-N-propargyl-3(2,4-dichlorophenoxy)- proylamine], reduces ROS production, which is thought to account for its neuroprotective function, suggesting that MAO-A may be an apoptogenic gene [32]. Currently, three inhibitors of type-B monoamine oxidase (MAO-B)—selegiline, rasagiline, and safinamide—are used for the treatment of Parkinson’s disease (PD) [33]. In light of this evidence, it appears that AChE and MAO are potential treatments of AD [30]. 

However, it has also been reported that dietary intake of polyphenol compounds attenuates oxidative stress and reduces the risk of developing related neurodegenerative diseases such as AD, Parkinson’s disease, stroke, multiple sclerosis, and Huntington’s disease [34]. Indeed, polyphenols and natural compounds can modulate various neurotransmitter systems in the brain; for instance, curcumin, which modulates serotoninergic and dopaminergic neurotransmission, acts as an antidepressant agent via regulating the levels of MAO-A and MAO-B enzymes [35,36]. So far, several studies exploring the ability of polyphenols in the management of neurodegenerative disorder and major depression [19] have been undertaken.

In this study, we explored novel biological properties of AFPE, investigating its effect on enzymes involved in brain functions and age-related disorders such as AChE, MAO-A, and MAO-B, using in vitro enzyme assays. Furthermore, we analyzed the effects of AFPE on the viability of human neuroblastoma SH-SY5Y and fibroblast BJ-5ta cell lines. Our results indicate that AFPE is able to reduce AChE, MAO-A, and MAO-B enzyme activity, thus indicating that it is a useful supplement for the prevention and treatment of neurodegenerative disorders.

## 2. Results

### 2.1. AFPE Total Polyphenolic Content

The total polyphenol content of the *Annurca* apple extract measured by Folin-Ciocalteu assay resulted in 1.26 ± 0.05 mg of catechin per g of apple flesh; this concentration was similar to that measured in other studies [11,15,17,37]. A HPLC analysis of AFPE (see Supplementary Figure S1) identified (–)-epicathechin, (+)-catechin, and chlorogenic acid as the *Annurca* apple *o*-diphenols.

### 2.2. Effect of AFPE on Cholinesterase Activity

**Figure 1:**
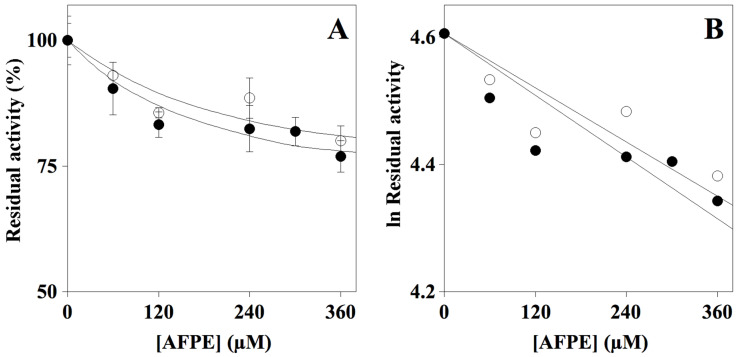
Effect of AFPE on cholinesterase activity. (**A**) AChE (filled circle) or BuChE (empty circle) was assayed in the absence or presence of the indicated amounts of AFPE, as reported in the Methods section. NB. 100 µM AFPE in the reaction mixture corresponded to 23 mg of apple flesh/mL. (**B**) Data were analyzed according to first order behavior, and the IC_50_ was calculated from the slope of the linear regression (r^2^, 0.924 for AChE and 0.910 for BuChE).

Currently, AChE inhibitors are one of the major classes of approved drugs to treat AD [38]; therefore, many research studies are still being devoted to the identification of novel agents with AChE inhibitory properties for the treatment of this neurodegenerative disease [26,27,28]. To study the effect of AFPE on cholinesterase activity, an in vitro enzyme assay [39] that allows an accurate determination of inhibition values was used. The assay was performed in the absence or presence of different AFPE concentrations, and the result (Figure 1, Panel A) indicated that AFPE exhibits both AChE and BuChE inhibitory activity, with a slight preference for AChE.

The analysis of the data by a semilogarithmic plot (Figure 1, Panel B) allowed us to determine the inhibitor concentration required for the inhibition of half of the activity (IC_50_), corresponding to 859 ± 18 µM and 966 ± 72 µM for AChE and BuChE, respectively. It should be noted that, under identical experimental conditions, donepezil, used as a positive control of inhibition, displayed an IC_50_ of 20 ± 0.82 nM and 3.5 ± 1.0 µM for AChE and BuChE, respectively (Supplementary Figure S2).

### 2.3. Effect of AFPE on MAO-A and MAO-B Enzyme Activity

**Figure 2:**
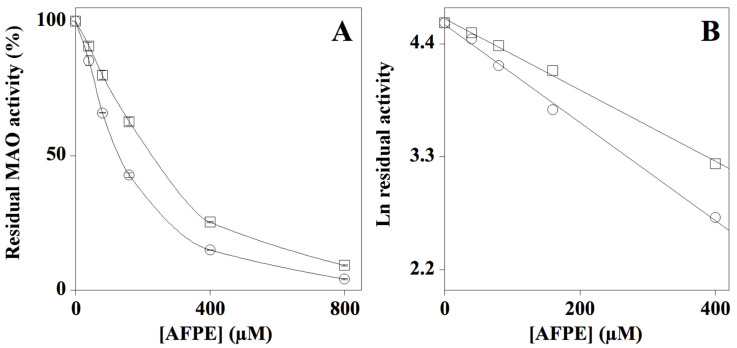
Effect of AFPE on MAO-A and MAO-B activity. (**A**) The MAO-A (circle) and MAO-B (square) enzyme activity was assayed in the absence or presence of the indicated amounts of AFPE. NB. 100 µM AFPE in the reaction mixture corresponds to 23 mg of apple flesh/mL. (**B**) Data were analyzed according to first order behavior, and the IC_50_ values were calculated from the slope of the linear regression (r^2^ = 0.998 for MAO-A and MAO-B).

MAO inhibitors are another group of approved drugs used to treat neurodegenerative diseases [32]. To study the AFPE effect on monoamino oxidase activity, an in vitro enzyme assay [40] was used to study the inhibitory activity of this extract. The assay was performed in the absence or presence of different AFPE concentrations. The result (Figure 2, Panel A) indicated that AFPE exhibits both MAO-A and MAO-B inhibitory activity, with a preference for MAO-A. In particular, an IC_50_ of 145 ± 3 µM and 199 ± 7 µM was calculated for MAO-A and MAO-B, respectively It should be noted that, under identical experimental conditions, clorgylin and selegiline, used as positive controls of inhibition for MAO-A and MAO-B, displayed an IC_50_ of 150 ± 22 nM and 230 ± 48 nM (Supplementary Figure S3).

### 2.4. Effect of AFPE Treatment on Viability and Morphology of Human Neuroblastoma SH-SY5Y and Fibroblast BJ-5ta Cell Lines

**Figure 3:**
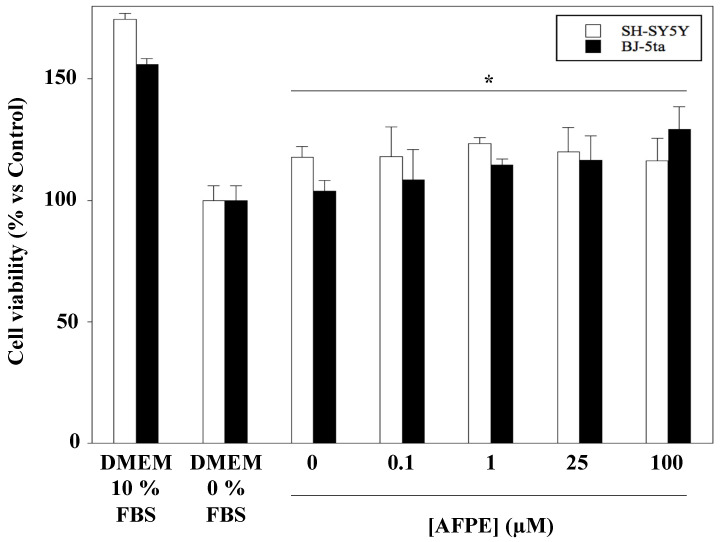
Effect of AFPE exposure on viability of SH-SY5Y and BJ-5ta cells. Cells kept in serum-free DMEM were treated with the indicated increasing amounts of AFPE in serum-free conditions for 24 h, and then subjected to an MTT assay. For each treatment, data are presented as mean ± SD (*n* = 3) of the control cells, cultured in serum-free DMEM, set as 100%. NB. 100 µM AFPE in the reaction mixture corresponds to 23 mg of apple flesh/mL. * Not significant vs. control cells (*p* values of 0.3345 and 0.1617 for SH-SY5Y and BJ-5ta cells, respectively).

The results of the enzyme inhibitory activity of AFPE prompted us to study its cytotoxicity in a neuronal derived cell line. With this aim, we examined the effect of AFPE on cell growth and morphology in human neuroblastoma SH-SY5Y and human fibroblast BJ-5ta cell lines by treating them with increasing amounts of the extract, in accordance with the literature [12]. Cell proliferation was analyzed by an MTT assay (Figure 3). The results indicated that exposure to increasing amounts of AFPE in serum-free conditions for 24 h did not affect cell viability, either in SH-SY5Y or in BJ-5ta cells. Moreover, in both cell lines, a slight increase in cell viability (about 10–20%) in AFPE treated vs. untreated cells was observed, in agreement with previous results obtained in NIH3T3 fibroblasts [41]. It should be noted that under our experimental conditions, the highest concentration of AFPE tested (100 μM) did not affect cell viability. In addition, increasing amounts of AFPE over 24 h did not significantly change the cell morphology of SH-SY5Y cells compared to that of untreated control cells kept in serum-free-DMEM, as evaluated by phase-contrast analysis (see Supplementary Figure S4). However, a moderate decrease in neurite-bearing cells was observed in cells treated with the highest AFPE concentration (100 μM), suggesting a possible modulation of cell phenotype, a finding already observed in other nervous cell lines [42,43].

## 3. Discussion

Polyphenols are a widespread group of secondary metabolites in plants, representing the most desirable phytochemicals due to their potential for use in the food industry, cosmetics, medicines, and other fields.

Several studies on dietary polyphenols have demonstrated their chemopreventive activities [44,45,46,47,48]. Moreover, polyphenols from *Annurca* flesh apple have been reported to exhibit antioxidant and antiproliferative properties in cancer cells [14,16]. The in vitro antioxidant power of polyphenolic extract has been evaluated, and AFPE has been found to have an even higher antioxidant value than vitamin C, a well-known nonpolyphenolic antioxidant. Moreover, AFPE exhibits the highest antioxidant power compared to a single polyphenol, (+)-catechin or (−)-epicatechin, probably due to the synergistic action that is created between the various polyphenols present in the mixture [11,49].

Polyphenols have been studied extensively for their antioxidant and anti-inflammatory activities, both of which are important in triggering the pathogenesis of different neurodegenerative diseases [20]. In fact, polyphenols, while targeting multiple targets, have been proven to be beneficial for the prevention of neurodegenerative disorders, and may delay the process of neurodegeneration [50]. For example, tea polyphenols have been extensively reported to confer protection against neurodegenerative diseases, including AD and Parkinson’s disease [51,52].

Polyphenols have several interacting groups which can form hydrogen bonds as well as weaker van deer Waals interactions; it is therefore hypothesized that they may inhibit the enzymes responsible for the pathophysiological processes underlying neurodegenerative diseases.

The inhibition of AChE is one of the therapies most commonly pursued in the treatment of mild to moderate AD. AChE is an enzyme that is responsible for the degradation of acetylcholine (ACh), and its inhibition results in increased acetylcholine levels, thus improving the cognitive symptoms of neurodegenerative diseases. The cholinergic hypothesis of AD postulates that degeneration of cholinergic neurons and the resultant decrease in the activity of AChE and depletion in the level of ACh are the immediate and consistent causes of the cognitive decline and dementia in neurodegenerative diseases. Polyphenols have been found to potentially inhibit both AChE and BuChE enzymes. Thus, it is speculated that this inhibition elevates the level of ACh in the brain and thereby ameliorates the behavioral abnormalities associated with neurodegenerative diseases. Therefore, *Annurca* flesh polyphenols could potentially interact with the active sites of key drug targets of neurodegenerative disease such as AD. In vitro studies have shown that quercetin, a plant flavonol from the flavonoid group of polyphenols, is a competitive inhibitor of AChE and BuChE because it inhibits both enzymes in a concentration-dependent manner [53]. Quercetin inhibits AChE, secondary to hydrophobic interactions and strong hydrogen bonding with the enzyme, reducing the hydrolysis of ACh and thus increasing ACh levels in the synaptic cleft. Studies have linked the presence/absence of OH groups on the phenyl rings of the test compound to the inhibition of AChE and BuChE, given that the OH groups form hydrogen bonds with amino acid residues at the active site of the enzyme. This phenomenon could explain the inhibitory efficacy of *Annurca* polyphenols for both of these enzymes [54].

These results demonstrate novel biological properties of AFPE: it inhibits, to varying extents, the activity of enzymes such as AChE, BuChE, MAO-A, and MAO-B, which are involved in neurodegenerative disorders. Furthermore, our results demonstrate that, in the same concentration range, AFPE did not affect the cell viability of the human neuroblastoma SH-SY5Y and fibroblast BJ-5ta cells.

Therefore, because these phytochemicals are capable of crossing the blood–brain barrier (BBB), they are potential agents for the prevention of neurodegenerative disorders; however, different polyphenols subclasses vary in their ability to cross the BBB. In the case of AD, their efficacy is attributed to reducing Aβ toxicity and decreasing oxidative stress [55]. Nevertheless, anti-AD effects of certain flavonoids, such as rutin, myricetin, catechins, fisetin, quercetin, kaempferol, and apigenin, have been reported [54].

Thus, *Annurca* flesh apple polyphenols could emerge as alternatives to the drugs currently used for the treatment of neurodegenerative diseases. Nevertheless, further in vivo and in vitro studies are warranted to establish the efficacy of polyphenols in this respect.

## 4. Materials and Methods

### 4.1. Materials

Acetylcholinesterase from *Electrophorus electricus* (AChE), butirrylcholinesterase from equine serum (BuChE), acetylthiocholine, butirrylthiocholine, 5′,5′-dithiobis-2-nitrobenzoic acid (DTNB), Monoamine oxidase A and B, kynuramine, donepezil, clorgylin, and selegiline were purchased from Sigma-Aldrich (Milano, Italy). Dulbecco’s modified Eagle medium (DMEM), fetal bovine serum (FBS), trypsin-EDTA, and phosphate-buffered saline (PBS) pH 7.4 were obtained from Lonza (Basel, Switzerland); 3-(4,5-Dimethyl-2-thiazolyl)-2,5-diphenyl-2*H*-tetrazolium bromide, MTT was obtained from Sigma–Aldrich (Milano, Italy).

### 4.2. Annurca Apple Flesh Polyphenol Extraction (AFPE) and Polyphenols Evaluation

*Annurca* apple (*Malus pumila* cv. *Annurca*) fruits (each weighing about 100 g) were obtained from farms in Giugliano (Napoli, Italy) in October, right after the fruit was harvested (green peel). Fruits were reddened in the “*melai*” (Supplementary Figure S5) according to a specific procedure and then sent to the market. Polyphenol extraction from *Annurca* flesh apples was carried out on 40 g of apple flesh as previously reported [37], using 40 mL of 80% methanol and 20% water containing 0.18 M HCl. The total polyphenolic content in the 65 mL extract obtained was assessed by the Folin-Ciocalteu colorimetric method [56] on 50–200 µL of the extract, and the absorbance reading was compared to a standard curve of catechin solutions [11,37]. The concentration of the extract was brought to a 10 mM catechin equivalent in PBS, corresponding to 2.3 g of *Annurca* Apple flesh per ml of extract. The AFPE was aliquoted and stored at –80 °C until use. The polyphenol composition of AFPE was assessed by HPLC as reported [37]. The main *o*-diphenols, identified on the basis of the retention time of the authentic standard, were (+)-catechin, (−)-epicatechin, and chlorogenic acid.

### 4.3. Cholinesterase Enzyme Inhibition Assay

AChE or BuChE activity was assayed by the Ellman method [39] using acetylthiocholine or butirrylthiocholine as substrate, respectively. The reduction of dithiobisnitrobenzoate by the thiocholine, produced by the enzymatic hydrolysis of thiolated substrates, was followed colorimetrically (412 nm) at room temperature (22–27 °C) using a Cary 100 UV-VIS Spectrophotometer (Agilent). Briefly, the reaction mixture (500 µL) contained: 330 µM 5,5′-dithio-bis-2-nitrobenzoic acid (DTNB), 500 µM acetylthiocholine or butirrylthiocholine as substrate, and different amounts of AFPE in 0.1 M sodium phosphate buffer with pH 7.4. The reaction was started by the addition of 100 mU/mL AChE or BuChE, and the initial rate of the reaction was derived from the linear portion of the kinetics. The concentration of AFPE required to reduce the enzymatic activity to 50% (IC_50_) was derived from semilogarithmic plots. Linear curve fits were obtained with the least-squares method, and the significance of the correlation was estimated from the squared correlation coefficient r^2^.

### 4.4. Monoamine Oxidase A and B (MAO-A and -B) Enzyme Inhibition Assay

A monoamine oxidase assay was carried out using a fluorimetric method as previously reported [40], in which the oxidation of kynuramine by monoamine oxidase led to the production of 8-hydroxychinoline, a compound which becomes fluorescent in alkaline conditions. The 250 µL reaction mixture was prepared in a 50 mM potassium phosphate buffer, pH 7.1, and contained 40 µM kynuramine in the absence or presence of the indicated concentration of the AFPE. The reaction was started by adding 3.75 µg monoamine oxidase A or B and allowed to proceed for 20 min. The enzymatic oxidation of the substrate was stopped by adding 150 µL of 2 M NaOH, and after 10 min incubation at room temperature, 240 µL of water. The resulting mixture was centrifuged for 10 min at 15,000 rpm and the fluorescence was read on 500 µL supernatant using a Cary Eclipse Spectrofluorimeter (Varian). The fluorescence signal was recorded at room temperature (20–25 °C) using an excitation and emission wavelength of 315 and 380 nm, respectively; slits were set to 10 nm, both for the excitation and the emission beam. The residual activity was referred to that measured in the absence of AFPE, and the data were collected in three different experiments. The concentration leading to 50% residual activity (IC_50_) was derived from a semilogarithmic plot in which the logarithm of the residual activity was plotted against AFPE concentration.

### 4.5. Cell Cultures and Treatments

The human neuroblastoma SH-SY5Y and human skin fibroblast BJ-5ta cell lines were cultured in Dulbecco’s Modified Eagle’s Medium (DMEM), supplemented with 10% heat-inactivated fetal bovine serum (FBS), 1.5 mM L-glutamine, 100 units/mL penicillin, and 100 μg/mL streptomycin under a humidified atmosphere of 5% CO_2_ at 37 °C. Subconfluent cells were plated (1 × 10^4^ cell/well) onto a 96-well plate and treated with increasing concentrations of AFPE [37] for 24 h in serum-free DMEM to determine the IC_50_ value. The treatments were performed under serum-free conditions.

### 4.6. Cell Viability Assay and Morphology Analysis

Cell viability was evaluated as mitochondrial metabolic activity [57] using the MTT assay as previously reported [10]. Briefly, the cells were plated onto 96-well plates (1 × 10^4^ cells/well), in DMEM with 10% FBS. After 24 h seeding, the cells were treated with different concentrations of AFPE or DMEM containing 0.2% PBS. After 24 h, 10 μL of the MTT solution (5 mg/mL) was added to each well in the dark, and the plates were incubated for 3 h at 37 °C under 5% CO_2_ atmosphere. Following medium aspiration, double washing with 100 µl PBS, and solubilization of formazan crystals with 250 µL DMSO, the absorbance was measured at a wavelength of 570 nm using an ELISA plate reader (BioRad, Milano, Italy). The cell viability was expressed as a percentage relative to the untreated cells, cultured in a serum-free medium, set as 100%.

To evaluate cell morphology, the cells were seeded subconfluently onto a 24-multiwell, treated with different AFPE concentrations or in DMEM containing 0.2% PBS, and then observed for 24 h by a phase-contrast Zeiss Axiovert 40 CFL inverted microscope (Carl Zeiss, Milan, Italy) using a LD A-Plan 10×/0.50 P h 2 objective and equipped with a 12.1-megapixel CCD digital capture camera (Canon, PowerShot G9, Italy). Images were acquired using digital image software (Remote Capture DC, Canon).

### 4.7. Statistical Analysis

All the assays were performed in triplicate, and the data were expressed as mean ± SD of at least three independent experiments performed. Statistical significance was assessed by One-way analysis of variance (ANOVA) with the Bonferroni correction as a post hoc test, using Kaleidagraph (4.1 version) software by Synergy. The significance was accepted for *p* < 0.05.

## 5. Conclusions

In conclusion, our results demonstrated that AFPE shows a potentially neuroprotective effect for its ability to inhibit AChE and MAO-A and -B activity, enzymes involved in AD. Although further investigations will be necessary to clarify the molecular and cellular mechanisms underlying these AFPE-induced biological effects, our data highlight the potential use of these extract as nutraceuticals in the treatment of AD. Although only a low dose of polyphenol administration can be achieved via AFPE consumption, its relevance was underlined in a recent study [58] that reported that the combination of antioxidant properties with the cholinesterase inhibitory power of some purified polyphenols may be useful in the treatment of AD symptoms. However, further studies on the bio-availability and bio-accessibility of polyphenols, and therefore of the AFPE, are also needed to develop strategies for the production of herbal/plant extracts as a source of functional foods. Therefore, the biological properties of AFPE could potentially be utilized, not just in the pharmaceutical field, but also in the food industry.

## Data Availability

Data available in a publicly accessible repository.

## Supplementary Materials

The following supplementary figures are available online at https://www.mdpi.com/1424-8247/14/1/62/s1; Supplementary Figure S1, HPLC profile of AFPE; Supplementary Figure S2. Positive control of AChE or BuChE inhibition by donepezil. Supplementary Figure S3. Positive control of Monoamine oxidase (MAO) inhibition by Clorgylin or Selegiline, specific inhibitors of MAO-A and MAO-B, respectively. Supplementary Figure S4: Effect of AFPE on morphology of human neuroblastoma SHSY-5Y cells. Supplementary Figure S5: *Annurca* apple “melaio” for fruits reddening.
