# Supplementary material for: Annurca Apple Polyphenol Extract Affects Acetyl- Cholinesterase and Mono-Amine Oxidase In Vitro Enzyme Activity
Source: Pharmaceuticals (Basel). 2021 Jan 14;14(1):62. doi: 10.3390/ph14010062 (PMC7828649; doi:10.3390/ph14010062)
Supplement: Supplementary file 1 [file pharmaceuticals-14-00062-s001.pdf]

Supplementary Figure 1

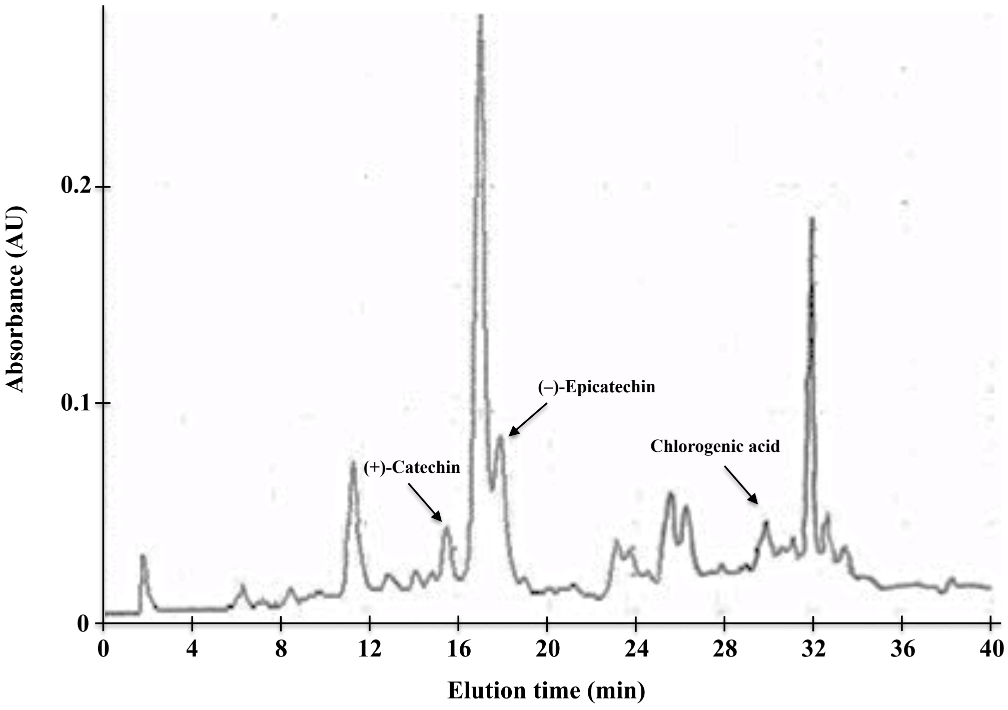

Supplementary Figure 1. HPLC profile of AFPE. HPLC separation of polyphenols was performed by reversed-phase chromatography on a 5 μm column Kromasil C18 column (150x4.6 mm), using a Beckman Apparatus (Gold-126) equipped with a UV detector fixed at 278 nm. The column was eluted at a flow rate of 1.0 ml/min with 0.2% acetic acid, pH 3.1 (A) and methanol (B) as the mobile phase; the gradient was changed as follows: 95% A/5% B for 1 min, 85% A/15% B in 1 min, 75% A/25% B in 20 min, 95%A/5%B in 3 min, and 0% A/100% B in 15 min. The main o-diphenols were identified on the basis of the retention time of authentic standard references: (+)-catechin, (–)-epicatechin, and chlorogenic acid.

Supplementary Figure 2

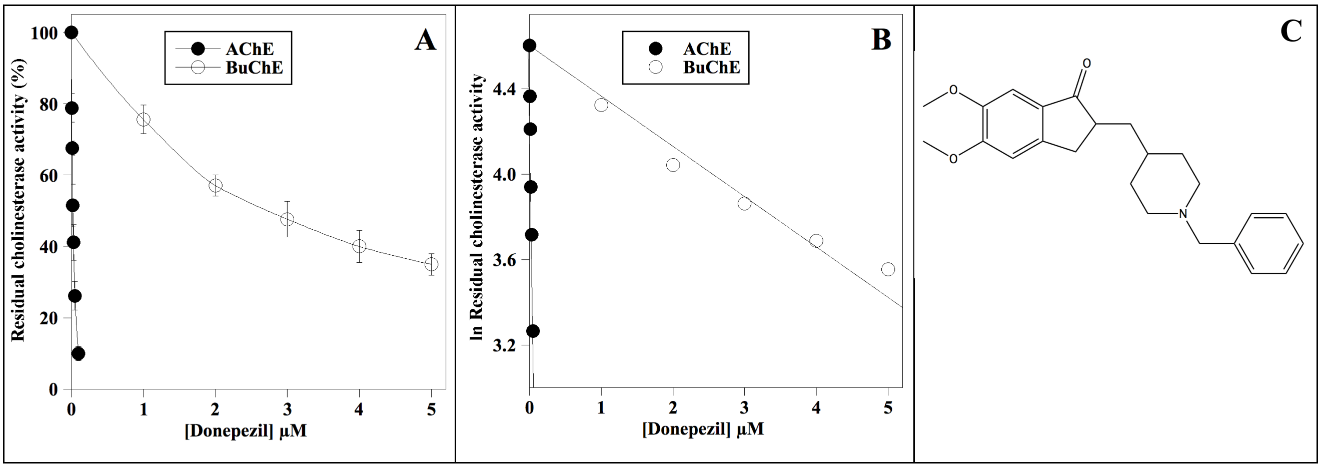

Supplementary Figure 2. Positive control of AChE or BuChE inhibition by donepezil. Panel A. Effect of donepezil on AChE and BuChE. The cholinesterase activity was determined kinetically by a colorimetric method in the presence of 100 mU/ml of electric ell AChE or serum horse BuChE using the appropriate thiolated substrate (acetyl-thiocholine or butyrril-thiocoline) in the presence of the indicate concentration of donepezil. The reaction was started by adding 330 μM DTNB. The standard deviation of the mean value of a triplicate determination, was indicated by bars.

Panel B. Semilogarithmic plot of the data reported in Panel A, for the determination of the IC_{50} value. The squared correlation coefficient of the regression displayed was 0.993 and 0.989 for AChE and BuChE inhibition, respectively.

Panel C. Donepezil structure obtained using the software ChemDraw, 12.0 version (CambridgeSoft, PerkinElmer, Waltham, MA, USA).

Supplementary Figure 3

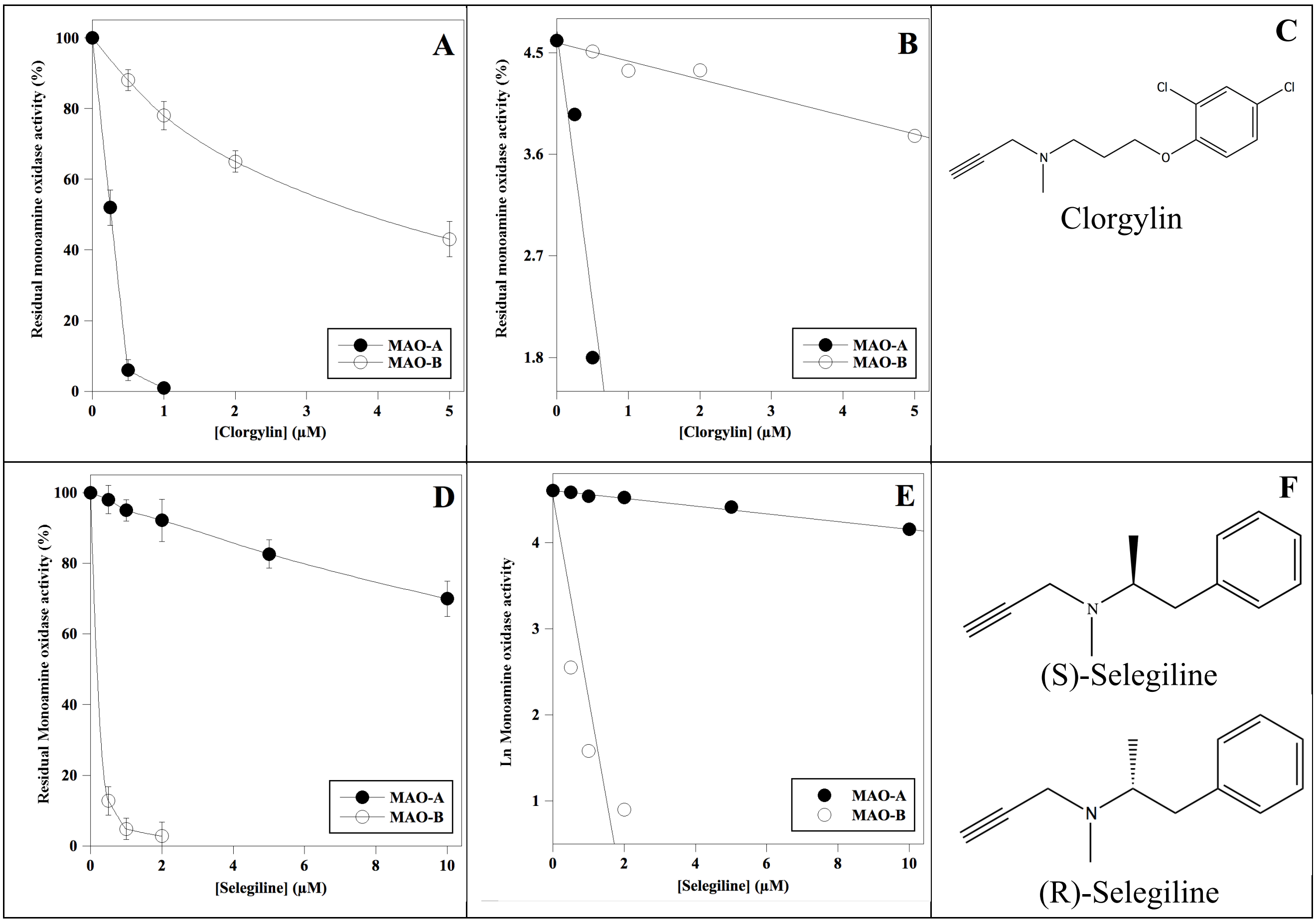

Supplementary Figure 3. Positive control of Monoamine oxidase (MAO) inhibition by Clorgylin and Selegiline, specific inhibitors of MAO-A and MAO-B, respectively.

Panels A and D. Effect of Clorgylin and racemic Selegiline on MAO activity. The activity was determined kinetically by a fluorimetric method using Kynuramine (40 µM) as substrate in the presence of 7.5 ng/ml of MAO-A (filled symbol) or MAO-B (empty symbol) at the indicate concentration of Clorgylin (Panel A) or racemic Selegiline (panel D). The standard deviation of the mean value of a triplicate determination, was indicated by bars.

Panels B and E. Semilogarithmic plot of the data reported in Panels A and D, respectively, for the determination of the IC_{50} value. The squared correlation coefficient of the regression displayed was never below 0.95.

Panels C and F. Clorgylin (Panel C) and (S)- or (R)-Selegiline (Panel F) structures obtained using the software ChemDraw, 12.0 version (CambridgeSoft, PerkinElmer, Waltham, MA, USA).

Supplementary Figure 4

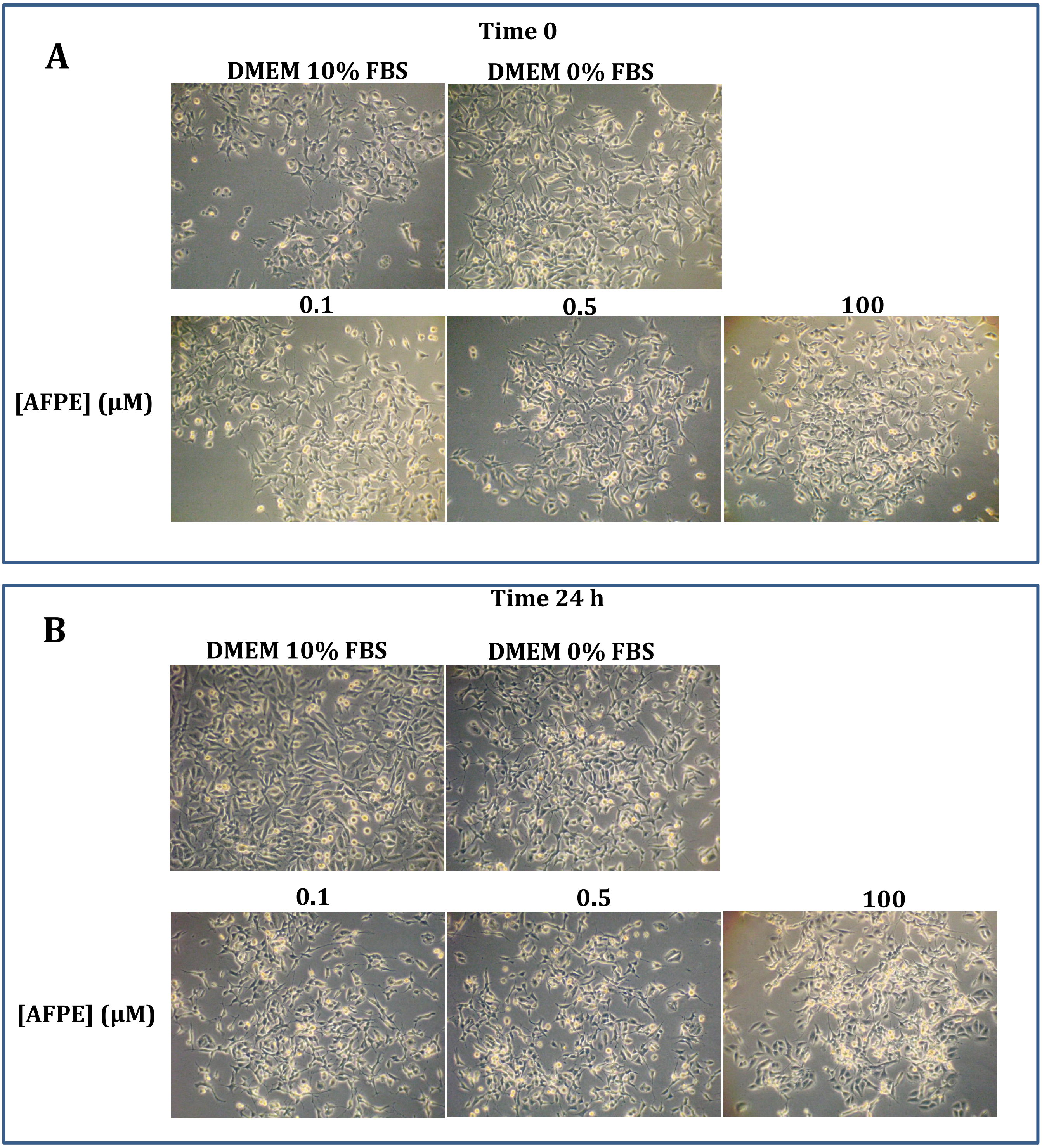

Supplementary Figure 4. Effect of AFPE on morphology of human neuroblastoma SH-SY5Y cells. Cells exposed to the indicated concentrations of AFPE for 24 h or kept in DMEM with 0.2% PBS, were observed at Time 0 (Panel A) and after 24 h (Panel B) by a phase-contrast Zeiss Axiovert 40 CFL inverted microscope using a LD A-Plan 10 ×/0.50 P h 2 objective and equipped with a 12.1-megapixel CCD digital capture camera. Images were acquired using digital image software. 100 µM AFPE corresponds to 23 mg of apple flesh/ml.

Supplementary Figure 5

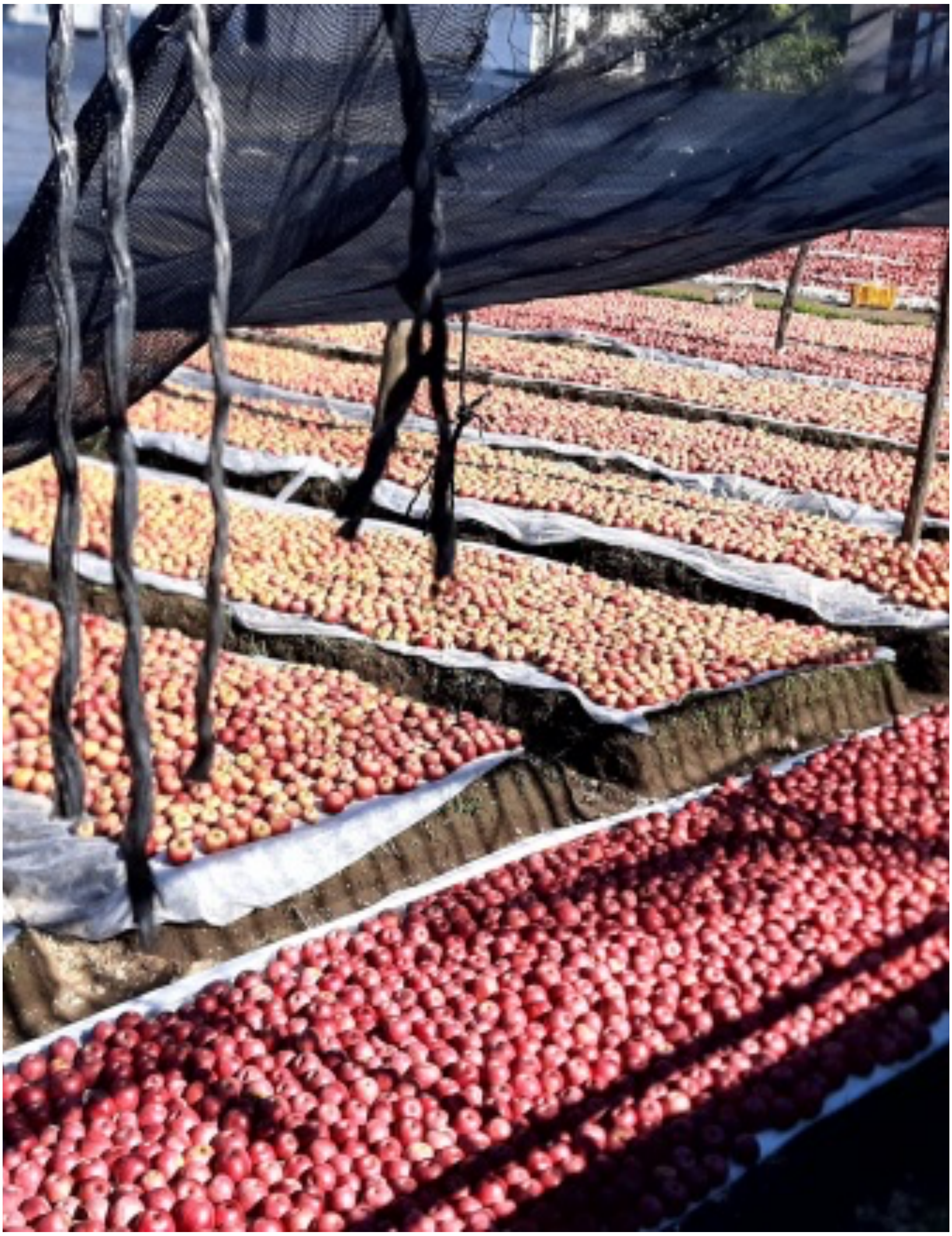

Supplementary Figure 5. Annurca apple "melaio" (month of October) for fruits reddening (Picture courtesy: Stefania D'Angelo)
